# Magnetic induction spectroscopy (MIS) as potential non-contact label-free technique for DNA amplification follow-up

**DOI:** 10.2478/joeb-2026-0011

**Published:** 2026-08-18

**Authors:** César A. González-Díaz, Amelia Laveille, Ulascan Yuce, Franck M. André, Lluis M. Mir

**Affiliations:** Instituto Politécnico Nacional, Escuela Superior de Medicina, México City, 11320, México.; Université Paris-Saclay, CNRS, Gustave Roussy, Biologie du cancer et Métabolisme, 94805, Villejuif,France.

**Keywords:** Biosensor, gene, magnetic induction, PCR

## Abstract

Gene amplification and detection techniques such as polymerase chain reaction (PCR) or quantitative PCR (qPCR) require the use of fluorescent reagents and probes or dyes. Both PCR and qPCR must be performed by trained personnel. The aim of this work was to assess the potential use of magnetic induction spectroscopy (MIS) as a non-contact, label-free and easy-to-use technique to detect gene amplification in final PCR products. We extracted Deoxyribonucleic Acids (DNA) from samples of mouse tissue and processed it for gene amplification through PCR. We considered six different biological samples for the exploration of four genotypes and their corresponding blank controls. In every sample, we carried out MIS measurements in two stages (before and after PCR) in the range of 0.073 to 1.125 MHz and compared the differences. The results indicate a MIS magnitude differential pattern in the range of 500 to 650 kHz, which warrants the potential use of the MIS technique for the development of a fully contactless, label-free biosensor and capable of detecting DNA directly from PCR products.

## Introduction

Detection of Deoxyribonucleic Acids (DNA) and genes is a key step in many molecular biology and molecular medicine applications. However, there is a critical need for a simple and affordable method for detecting amplified DNA through PCR, particularly in developing countries. Current methods include electrophoresis, fluorescence, and the recently developed electrochemical impedance spectroscopy (EIS), among others. EIS is a label-free analysis that uses a portable PCR system with a modified heating module that includes additional electrodes. It determines the presence of DNA based on electrochemical properties in a frequency range of 100 Hz to 1 MHz. In the PCR sample, EIS measurements showed a consistent trend and a significant change in impedance: the real and imaginary parts decreased with frequency and increased with the number of PCR cycles, proving the benefits of these measurements [1].

Another recently reported approach for nucleic acid quantification uses multifrequency impedance cytometry in a microfluidic chip and machine learning [2]. It involves a neural network based on a hybrid regression model to predict DNA quantities coupled to paramagnetic beads by leveraging electrical measurements in a frequency range of 100 kHz to 20 MHz. The real and imaginary parts of the maximum intensity within a microfluidic channel served as inputs for deep learning models. The results indicate its capability to utilize integrated impedance data to predict concentrations of DNA molecules coupled to the beads.

Label-free DNA detection and quantification by electrical bioimpedance spectroscopy (EBiS) combined with inter-digitated microelectrodes arrays was previously proposed and reported [3,4,5,6,7]. A relevant correlation between EBiS and DNA concentration has been documented at specific frequency ranges [3,4,5,6,7], showing the feasibility of using EBiS as a minimally invasive technique without any labeling or surface functionalization step. Although label-free DNA detection through microelectrodes with minimal sample contact is critical for simple and low-cost biosensors, a contactless DNA sample biosensor design remains a significant challenge, mainly to avoid capacitive effects due to the microelectrode-analyte interface and unspecific measurements. Indeed, under the influence of an electric field, the ions in the tested solutions tend to move towards the electrode-sample interface, forming ionic double layers at the interface. These double layers induce parasitic high capacitances in series to the conducting bulk of the sample. This phenomenon manifests as a high apparent dielectric constant, and the effect is known as electrode polarization (EP) [8].

Magnetic induction spectroscopy (MIS) is an emerging technique to determine the viability and functionality of biological samples [9,10,11,12,13,14]. A magnetic field is applied by a current-carrying primary coil to induce current in the analyte, which is then detected in a secondary coil. The potentials induced in the sensor coil are compared against the inductor coil in terms of magnitude and phase. MIS avoids errors associated with EP, which offers significant advantages. However, it might face other sources of error, such as the noise from inductive and resonant artifacts.

A previous theoretical modeling study demonstrates that MIS can distinguish bulk DNA concentrations through non-contact electromagnetic measurements [15]. In this study, we assess the potential use of MIS as a non-contact, label-free, and easy-to-use technique to detect nucleic acids amplification in final PCR products.

## Materials and methods

**Fig. 1:**
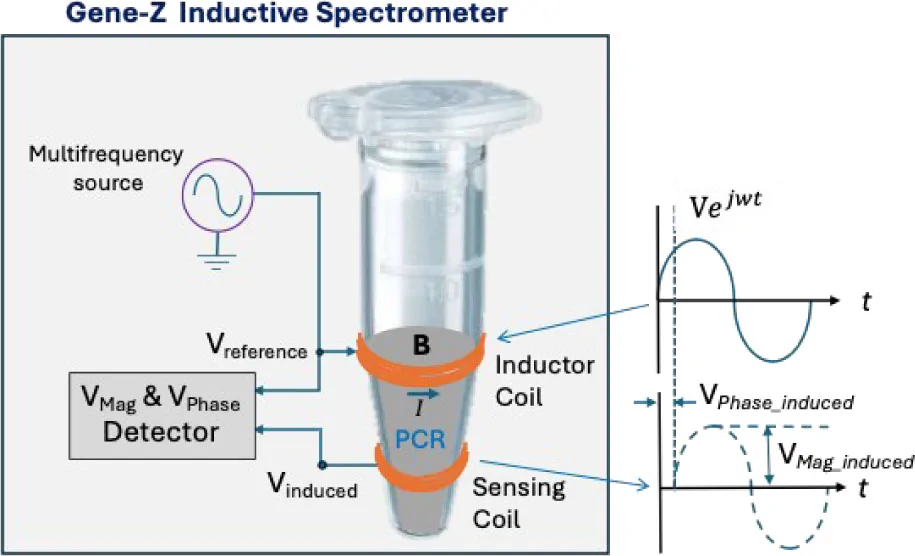
Schematic representation of the Gene-Z Inductive Spectrometer. The basic elements of the non-contact and label-free DNA biosensor design include a multifrequency voltage-controlled current source and a voltage magnitude (V_Mag_) and phase angle (V_Phase_) detector between two signals (reference vs. induced).

The proposed biosensor design consists of a system for magnetic field induction through two aligned coils with the analyte coaxially centered. The system is integrated by a multi-frequency function generator, an array of two inductor-sensor coils with signal coupling, and a magnitude and phase comparator between the inductive magnetic field and the detected field (Figure 1).

The induced magnetic field depends on the electrical properties of the sample. The magnetic field is perturbed, as evidenced by differences in the magnitude and phase of the potential detected in the inductive and sensor coils. These parameters are correlated with the characteristics of the analyte, providing valuable information to determine its relationship with the presence of DNA amplicons.

### Prototype Design

The prototype consists of five stages: control, function generator, inductor-sensor, gain and phase detector, and analog-to-digital converter. The system uses a micro-controller programming system and a user graphical interface. Control: Raspberry Pi 3 B+ was used to control the system using the Serial Peripheral Interface (SPI) communication protocol through the General-Purpose Input/Output (GPIO) ports. Function Generator: XR2206 Digital Synthesizer was used to produce an adjustable sinusoidal signal in the range of 0.07 to 1.2 MHz which is injected into the inductor-sensor stage, programmed trough a digital MCP42100 potentiometer. Inductor-Sensor: Inductive coils were designed with 32-gauge magnet wire, each coil was built with 5 turns with diameter of 5 and 3 mm for inductive and sensing respectively, separation between the coils is 3 mm. The sample for measurements is placed between the two coils in a typical 200 μL PCR tube. Gain and Phase detector: AD8302 Comparator was used to compare the phase shift and gain of the magnetic field induced in the sample compared to the initial field, obtaining two voltage signals corresponding to gain and phase. Analog-to-Digital Converter: MCP3008 was used to convert the analog voltage values (magnitude and phase) from the AD8302 comparator into digital values.

### DNA extraction and molecular assays

We extracted genomic DNA from mouse ear biopsies using the Platinum™ Direct PCR Universal Master Mix kit (Cat. A44647500, Invitrogen, Thermo Fisher Scientific, Waltham, Massachusetts, USA), following an adapted lysis protocol. We incubated each biopsy overnight at 55 °C under agitation in 306 μL of lysis solution (300 μL of lysis buffer and 6 μL of proteinase K, both supplied with the kit). We used crude lysates directly as PCR templates without further purification, in accordance with the kit’s compatibility with unpurified samples.

We amplified six DNA samples for four specific loci: The four loci selected for genotyping serve as reporters or drivers of Cre/Dre recombinase activity in distinct myeloid cell populations, enabling lineage tracing and conditional gene editing strategies. The Rosa26-tdTomato locus is a widely used Cre-reporter allele that drives tdTomato expression upon recombination, allowing permanent labeling of targeted cell populations. The LysM-Cre × CD36flox system enables myeloid-specific deletion of CD36, a scavenger receptor involved in lipid uptake and metabolic reprogramming. The two remaining loci involve inducible Cre-ERT2 and Dre-ERT2 recombinase systems restricted to specific tissue-resident macrophage subsets, allowing temporally controlled fate-mapping experiments. Each PCR (11 μL final volume) contained 5 μL of 2X Platinum™ Direct PCR Universal Master Mix, 0.5 μL of each primer, 3 μL of nuclease-free water and 2 μL of lysate. Each run included a positive control of known genotype (Cre^+^) and a no-template (H_2_O) negative control. We performed the amplification on a thermocycler with a heated lid set at 110 °C, using the following program: initial denaturation at 94 °C for 2 min; 35 cycles of 94 °C for 15 s (denaturation), 60 °C for 15 s (annealing), and 68 °C for 20 s (extension); and a final hold at 4 °C. The universal 60 °C annealing temperature is recommended by the manufacturer and obviates primer-pair-specific optimization.

We discriminated wild-type (WT) and mutant alleles by amplicon size using primer sets designed accordingly. We resolved PCR products (6 μL per sample) by electrophoresis on a 1.5 % agarose gel prepared in 1X TAE buffer (diluted from a 50X stock) and stained with SYBR™ Safe (2 μL per 100 mL of gel), alongside a GeneRuler Mix Range (Thermo Fisher Scientific) molecular weight marker for amplicon size estimation.

### MIS measurements

We carried out MIS measurements in the range of 0.073 to 1.125 MHz for every sample at both before and after PCR stages, and we compared the differences. We measured every sample five times and analyzed average values ± standard errors. To minimize potential external interference, samples and controls were treated under identical conditions; the MIS system being housed in a properly grounded Faraday cage. We performed the data analysis using MATLAB (MathWorks, Natick, MA, USA) licensed through MathWorks on-line for “Instituto Politécnico Nacional-México”.

### Ethical approval

The research related to animals use was conducted in accordance with all the relevant national regulations and institutional policies for the care and use of animals.

## Results

**Fig. 2:**
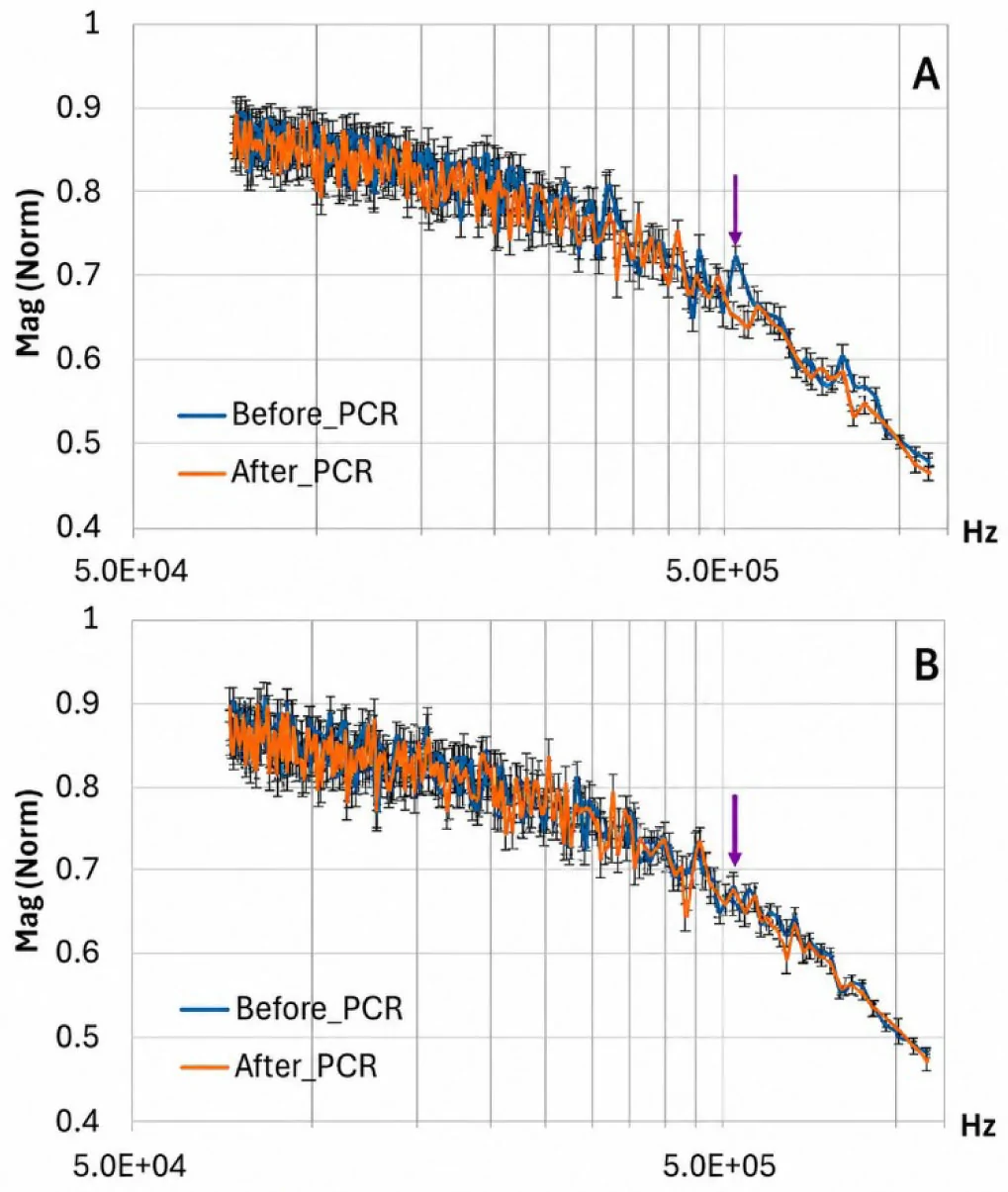
Spectra of MIS magnitude before and after PCR in A) positive gene amplification and B) blank controls. Data are presented as a pool of the average value of the six samples analyzed (six biological replicates × five technical repeats measurements each), thus every spectrum is a total of 30 measurements per point (at each frequency). Bars represent the standard error, and arrows show the differential pattern in the range of 500 to 650 kHz.

We normalized MIS data relative to the highest value in every spectrum for further analysis: such procedure was intended to neutralize the effect of inductive changes relative to sample positioning and inductive artifacts that might be evident in absolute values. Figure 2 shows the normalized spectra of the MIS magnitude (V_Mag_). Data are presented as a pool of the average value of the six samples analyzed (six biological replicates × five technical repeats measurements each), thus every spectrum is a total of 30 measurements per point (at each frequency) before and after PCR in two cases: positive gene amplification and blank controls. A differential pattern clearly emerges in the range of 500 to 650 kHz and it’s highlighted by arrows. No significant sensitivity was observed in the (V_Phase_) parameter.

### Area under the curve (AUC)

As a single quantitative descriptor of the overall spectral response across the observed differential pattern, we calculated the AUC in the sensitive range V_Mag_ (500–650 kHz) for each individual MIS spectrum by numerical integration of the spectral response as a function of frequency, In this context, the AUC descriptor represents the integral of the spectral magnitude and is intended for a preliminary feature extraction, with the aim of laying the groundwork for future classification analyses. For each sample, we integrated the spectral values within the selected frequency range using the trapezoidal numerical method implemented in MATLAB through the **trapz** function:

(1) $$AUC=\sum_{i=1}^{n-1}\frac{\left(y_{i}+y_{i+1}\right)}{2}(x_{i+1}-x_{i}),$$

Where *x_i_* is the frequency, *y_i_* is the spectral value, and *n* the number of frequencies analyzed.

### Statistics analysis

For every independent biological sample (*n* = 6) the AUC was estimated five times before and after PCR reaction. The five technical replicates were averaged to obtain a single representative value for each biological sample. The resulting paired observations were compared using a two-sided Wilcoxon signed-rank test. Because only one pre-specified paired comparison was performed, no correction for multiple comparisons was applied. Statistical significance was set at *p* < 0.05. We developed the statistical analysis using the IBM SPSS Statistics software ver. 31.0.2.0 (IBM Corp., Armonk, NY, USA). The paired difference was summarized by its median, and a 95% confidence interval was estimated using the bias-corrected and accelerated (BCa) bootstrap method [16].

The paired comparison showed a statistically significant difference between before and after PCR (Wilcoxon signed-rank test, Z = −2.201, p = 0.028, effect size r = 0.89), indicating that the PCR treatment produced a large effect [17,18]. The median paired difference was −2637.4 units (95% bootstrap BCa confidence interval: −5100.3 to −1428.7 units), indicating a large reduction after PCR.

**Fig. 3:**
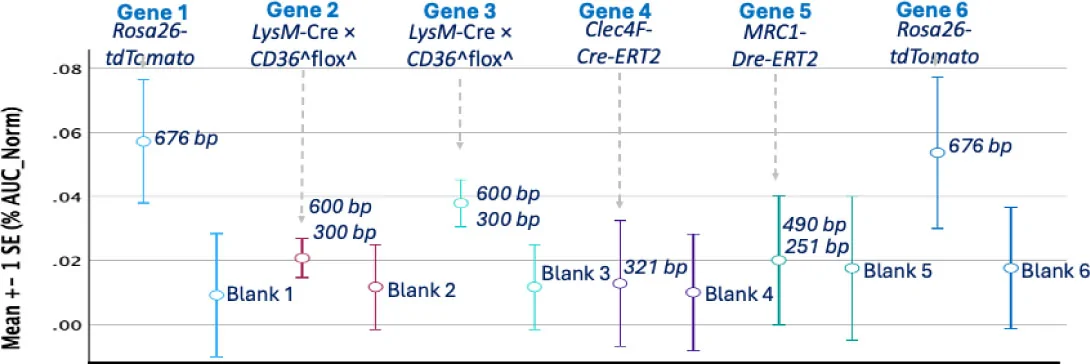
Individual %AUC change as parameter comparison. Differences after PCR with respect to basal condition (before PCR) for positive genes and corresponding blanks are showed. Molecular size data estimated by electrophoresis are shown for every amplicon’s presence in contrast with control blanks. A paired comparison for every independent biological sample (n = 6) showed a statistically significant difference before and after PCR (Wilcoxon signed-rank test, Z = −2.201, p = 0.028, effect size r = 0.90). (95% bootstrap BCa confidence interval: −5100.3 to −1428.7 units).

We grouped AUC values according to the experimental conditions: Figure 3 shows the individual ratio of change of the AUC as a percentage (%AUC) after PCR relative to the basal condition (before PCR) for every amplicon and corresponding blank. We include the molecular sizes observed in the images of samples electrophoresis in agar gel illuminated under UV, such data being also listed in the last column of Table 1.

**Table 1.** Gene samples: expected and observed molecular sizes by electrophoresis.

**Sample number**	**Gene**	**Expected molecular size (bp)**		**Observed molecular size (bp)**	
1,6	*Rosa26*-tdTomato	WT	676	WT	676
		Mutant	627		
2,3	*LysM-*Cre×*CD36*^flox^	WT	600	WT	600 *
		Mutant	300	Mutant	300 *
4	*Clec4F*-Cre-ERT2	WT	532	Mutant	321
		Mutant	321		
5	*MRC1*-Dre-ERT2	WT	490	WT	490*
		Mutant	251		251*

* Heterozygosity (presence of WT and mutant alleles).

## Discussion

The sensitivity observed in the magnitude of the induced potential around 500 kHz is consistent with previous experiments conducted by our research group to detect low concentrations of total DNA using MIS (496 kHz).

The localized increase in magnitude response observed between 500 and 650 kHz prior to PCR can be attributed to a frequency-dependent modification of the electromagnetic coupling between the two coils. The PCR process entails a substantial increase in double-stranded DNA content, which can alter the effective dielectric properties, ionic interactions, and electromagnetic losses of the sensing medium. These changes could modify volumetric impedance conditions and reduce the quality factor of the coupled-coil system, resulting in an attenuation of the resonance magnitude. Consequently, the pre-PCR peak may represent a state closer to the intrinsic resonance of the coil-sample system, whereas the presence of amplified DNA partially disrupts this coupling.

The differential pattern in the %AUC appears to be associated with the molecular size of the amplicons. The presence of amplicons of different lengths or molecular sizes changes the viscosity (η) of the medium. Since η can be expressed as a function of ionic mobility (μ) from the combination of Stokes' law and the electrophoretic mobility theory [19, 20], the reduction in the magnitude of the induced potential due to the presence of amplicons could be associated with a viscosity change effect. Interestingly, the simultaneous presence of WT and mutant alleles (heterozygosity) might promote a nonlinear effect on the change in %AUC.

At this stage the findings interpretation is speculative and supported by electromagnetic and electrochemical theory. Furthermore, observations so far suggest that MIS measurements are sensitive enough to detect the presence of amplicons. However, a specific assessment of sensitivity and specificity using a larger number of samples is required to confirm reproducibility, as well as the inclusion of more robust controls such as purified amplicons at known concentrations and/or serial dilutions were not explored in our study. The absence of these conditions represents a limitation of the present study, as they were not in the scope of the present report.

**Fig. 4:**
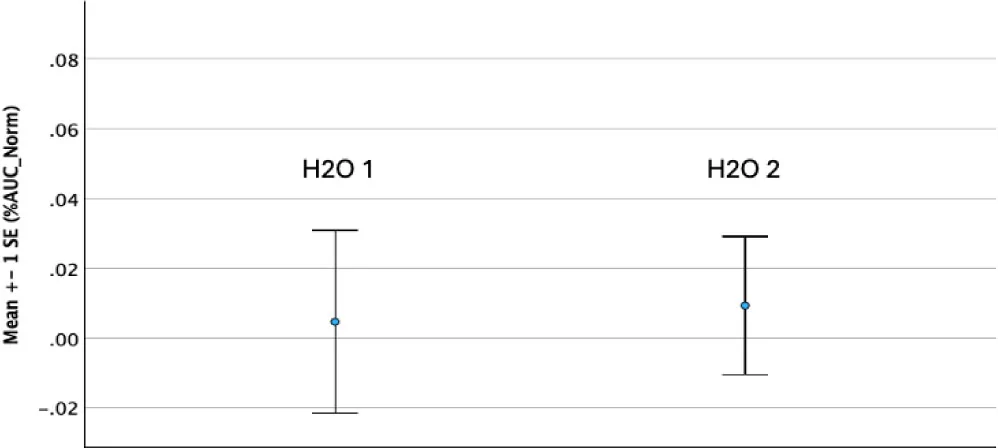
Individual %AUC change as parameter comparison. Differences after temperature cycles as PCR reaction simulation with respect to basal condition (before PCR) for two distilled water (H_2_O) samples.

The final PCR products generally show a decrease in volume compared to the initial condition due to the natural evaporation process; even if the decrease is minimal, it could certainly influence MIS observations. As control assay, two distilled water samples (H_2_O) were used to evaluate the evaporation effect through the used PCR temperature cycles set as a PCR reaction simulation. Figure 4 shows the individual ratio of change of the AUC as a percentage (%AUC) after PCR relative to the basal condition (before PCR) for every H_2_O sample. The findings suggest a clear basal MIS effect as a function of the temperature cycles evaporation, these conditions could explain the minimal change in %AUC observed in the blank samples (fig. 3), since no effect is expected in the absence of amplicons. Furthermore, blank control measurements showed no resonance peak, making external interference artifacts unlikely. However, coupling and parasitic effects inherent to the MIS system could still influence the observed signal, thus reducing electronic noise remains an area for improvement in our biosensor development.

## Conclusion

Spectra of MIS magnitude (V_Mag_) show a differential pattern in the range of 500 to 650 kHz and suggest the potential value of MIS as future view for the development of a fully contactless, label-free DNA biosensor capable of detecting genes directly from PCR products. The findings of this study suggest that the size of amplicons is important when detecting DNA amplification. We warrant additional experiments to confirm and to extend the observations.
